# Prevalence of Third Molar Agenesis: Associated Dental Anomalies in Non-Syndromic 5923 Patients

**DOI:** 10.1371/journal.pone.0162070

**Published:** 2016-08-31

**Authors:** Mamun Khan Sujon, Mohammad Khursheed Alam, Shaifulizan Abdul Rahman

**Affiliations:** 1 Orthodontic Unit, School of Dental Science, Universiti Sains Malaysia, Kubang Kerian, 16150, Kota Bharu, Kelantan, Malaysia; 2 Oral and Maxillofacial Surgery, School of Dental Science, Universiti Sains Malaysia, Kubang Kerian, 16150, Kota Bharu, Kelantan, Malaysia; Navodaya Dental College and Hospital, INDIA

## Abstract

The aim of this study was to investigate the prevalence of third molar agenesis and other associated dental anomalies in Bangladeshi population and to investigate the relationship of other dental anomalies with the third molar presence/agenesis. A retrospective study was performed using panoramic radiographs of 5923 patients, who ranged in age from 10 to 50 years. All radiographs were analyzed by Planmeca Romexis® 3.0 software (Planmeca Oy, Helsinki, Finland). Pearson chi-square and one way ANOVA (Post Hoc) test were conducted. The prevalence of third molar agenesis was 38.4%. The frequency of third molar agenesis was significantly higher in females than males (*p* <0.025). Third molar agenesis was significantly more prevalent in maxilla as compared to mandible (*p* <0.007). The prevalence of other dental anomalies was 6.5%, among them hypodontia was 3.1%. Prevalence of third molar agenesis varies in different geographic region. Among the other dental anomalies hypodontia was more prevalent.

## Introduction

Tooth agenesis is the congenital lack of one or more deciduous or permanent teeth, it also means the tooth which has not erupted in the oral cavity and is not visible in a radiograph [1]. Nowadays, approximately 50% of the third molars present some form of anomaly, either they remain un-erupted or partially erupted or they are absent from the oral cavity [2]. Its time of formation, and crown and root morphology are highly variable [3].

Environmental factors, genetic polymorphisms, systemic diseases, dietary habits and masticatory function can play an etiological role in the occurrence of dental anomalies like- microdontia, macrodontia, ectopic tooth eruption or agenesis[4–9]. Agenesis has been reported as the most frequently occurring dental anomaly [10]. Difference in sampling methods, research tools, source population, age and sex could explain the variations in reporting the prevalence of these anomalies [11]. According to a previous study, 12.7% prevalence of third molar agenesis was reported in British population [12]; Gracia-Hernandez et al. [13] found a prevalence of 24.75% in Chile population and Lee et al. [14] reported 41% for Korean population. Alam et al. [11] reported 30% and 33% prevalence in Malaysian Malay and Malaysian Chinese, respectively. Jacob et al. [15] found that the most frequent pattern of third molar agenesis was 18 > 28 > 38 > 48. However, Alam et al. [11] reported a higher agenesis rate of 48 than the 38.

Third molar agenesis has been associated with dental numeric and morphological variations. Garn et al. [16] reported that with the absence of a third molar, agenesis of the remaining teeth 13 times more likely. Third molar agenesis has also been associated with delayed development of certain teeth, reduced tooth size and morphology [17–19]. Due to geographical variations in the prevalence of third molar agenesis, we intend to investigate the prevalence of third molar agenesis and other dental anomalies in Bangladeshi population for the first time. Hence, the aim of this study was to investigate -

1. the prevalence of third molar presence/agenesis.
2. difference in sexes.
3. difference between maxilla and mandible.
4. the prevalence of other dental anomalies.
5. association of third molar presence/agenesis with other dental anomalies.

## Material and Methods

Panoramic radiographs of 5923 patients (2835 female and 3088 male) attending two renowned diagnostic centers of Dhaka, Bangladesh during the period from February, 2014 –February, 2015 were collected from the archive randomly.

### Inclusion Criteria

1. Age ranged from 10 to 50 years.
2. Patients who had not undergone surgical removal or extraction of any tooth.
3. Only vivid radiographs are included.

### Exclusion Criteria

1. Patients with congenital disorders.
2. Patients with facial clefts or other craniofacial deformity.
3. Radiograph which shows pathologies like- tumor, cyst etc.
4. Poor quality radiograph.

Based on inclusion and exclusion criteria finally 4228 (2092 female and 2136 male) radiographs were selected for this study. Two caliber investigators examined the radiographs using Planmeca Romexis® 3.0 software (Planmeca Oy, Helsinki, Finland). Data were collected from the diagnostic center’s archive with prior permission from the authority for research purposes only. Research related work was conducted in School of Dental Science, Hospital Universiti Sains Malaysia (HUSM). This study was approved by the Ethical Committee of the HUSM (USM/JEPeM/15080273), which complies with the Declaration of Helsinki. Patient records/information was anonymized and de-identified prior to analysis and kept confidential. This study was designed and conducted according to the Strengthening the Reporting of Observational studies in Epidemiology (STROBE) guidelines. Following dental anomalies were investigated-

#### Tooth Agenesis

It is the congenital lack of one or more deciduous or permanent teeth, it also means the tooth which has not erupted in the oral cavity and is not visible in a radiograph [1].

#### Hypodontia

Congenital absence of 1 to 5 teeth excluding third molar [20].

#### Hyperdontia

An increase in number of teeth in relation to the normal dental formula [21].

#### Impacted Canine

Tooth impaction is a pathological condition in which a tooth cannot or will not erupt into its normal functioning position, unless facilitated by treatment [22].

#### Peg Shaped Maxillary Lateral Incisors

It is a condition where mesiodistal crown width of a lateral incisor is smaller compared with the same dimension of the opposite mandibular lateral incisor of the same patient [23].

#### Microdontia

It is a condition in which one or more permanent teeth appear smaller than normal size [24].

#### Dilaceration

It is a developmental disturbance in shape of teeth. It refers to an angulation, or a sharp bend or curve, in the root or crown of a formed tooth [25].

### Statistical Analysis

Data were statistically analyzed by using IBM Statistical Package for Social Science (SPSS), Version 22.0 (SPSS, Chicago, IL, USA). The prevalence of third molar agenesis/presence and other dental anomalies were calculated by the Pearson chi-square and also one way ANOVA (Post Hoc) test were used for multiple comparisons between the groups. A *p* value of <0.05 was considered statistically significant.

## Results

### Prevalence of third molar agenesis

Table 1 shows 1624 subjects had at least one third molar missing among the 4228 subjects examined, thus the prevalence was 38.4%. Moreover, prevalence of one third molar missing was higher among others. The frequency of number of third molar agenesis was 1 > 2 > 4 > 3.

**Table 1:** Distribution of subjects with third molar presence/agenesis in Bangladeshi population.

Sex	n	0	1	2	3	4	n^a^
**Male**	2136	1351 (63.2)	338 (15.8)	277 (13.0)	81 (3.8)	89 (4.2)	785 (36.8)
**Female**	2092	1253 (59.9)	347 (16.6)	288 (13.8)	93 (4.4)	111 (5.3)	839 (40.1)
**Total Sample**	4228	2604 (61.6)	685 (16.2)	565 (13.4)	174 (4.1)	200 (4.7)	1624 (38.4)

All the counts are by number of patients. n = number of patients, 0 = patients have no agenesis, 1 = patients with agenesis of 1 third molar, 2 = patients with agenesis of 2 third molars, 3 = patients with agenesis of 3 third molars, 4 = patients with agenesis of 4 third molars. n^a^ = patients with at least one third molar missing.

### Sex Disparities

Table 2 represents the distribution and prevalence of third molar agenesis according to sex. Frequency of third molar agenesis was higher in females than males. The prevalence of third molar agenesis for females and males were 40.1% and 36.8% respectively.

**Table 2:** The frequency of agenesis according to sex.

Sex	n	Agenesis	Prevalence	X^2^	*p* value
**Male**	2136	785	36.8%	5.02	.025*
**Female**	2092	839	40.1%		

* p value <0.05 is significant

### Jaw Disparities

**Fig 1:**
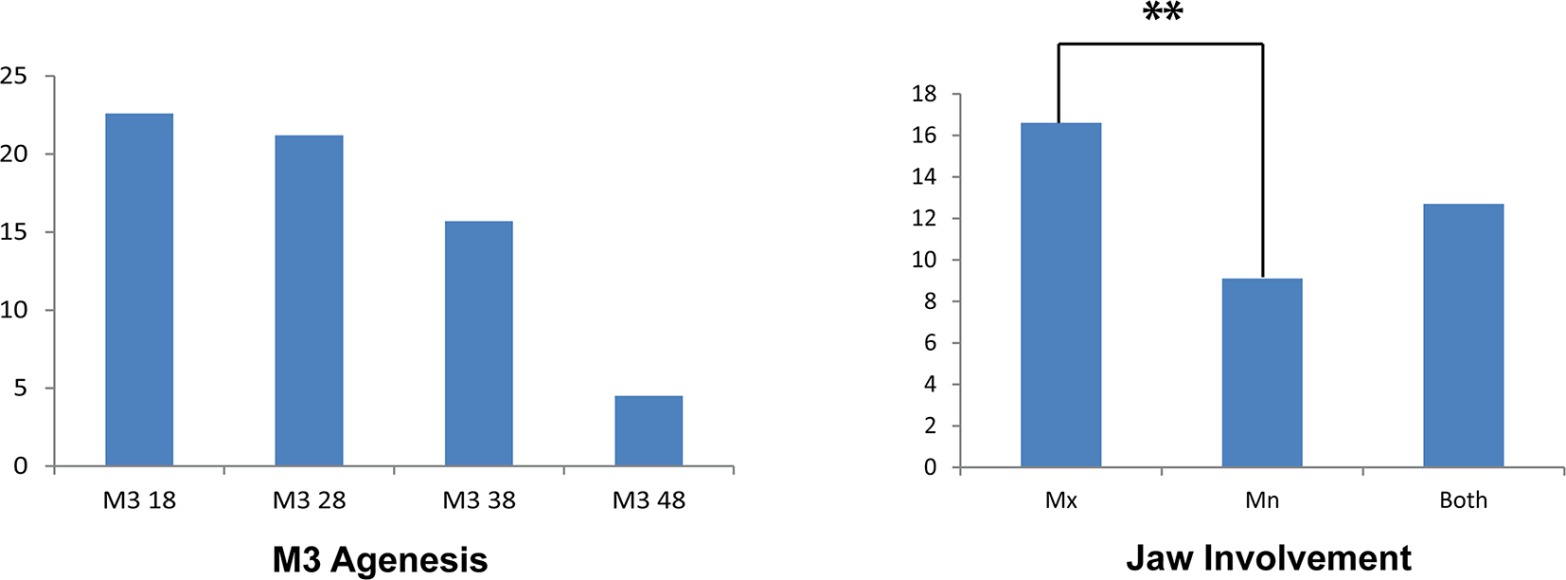
Distribution of subjects in total population with third molar agenesis and jaw involvement.

Fig 1 shows the most frequent pattern of third molar agenesis was 18>28>38>48. Third molar agenesis had a greater predilection for the maxillary arch than the mandibular arch, and the difference was statistically significant (*p* value = .007).

### Side Disparities

**Fig 2:**
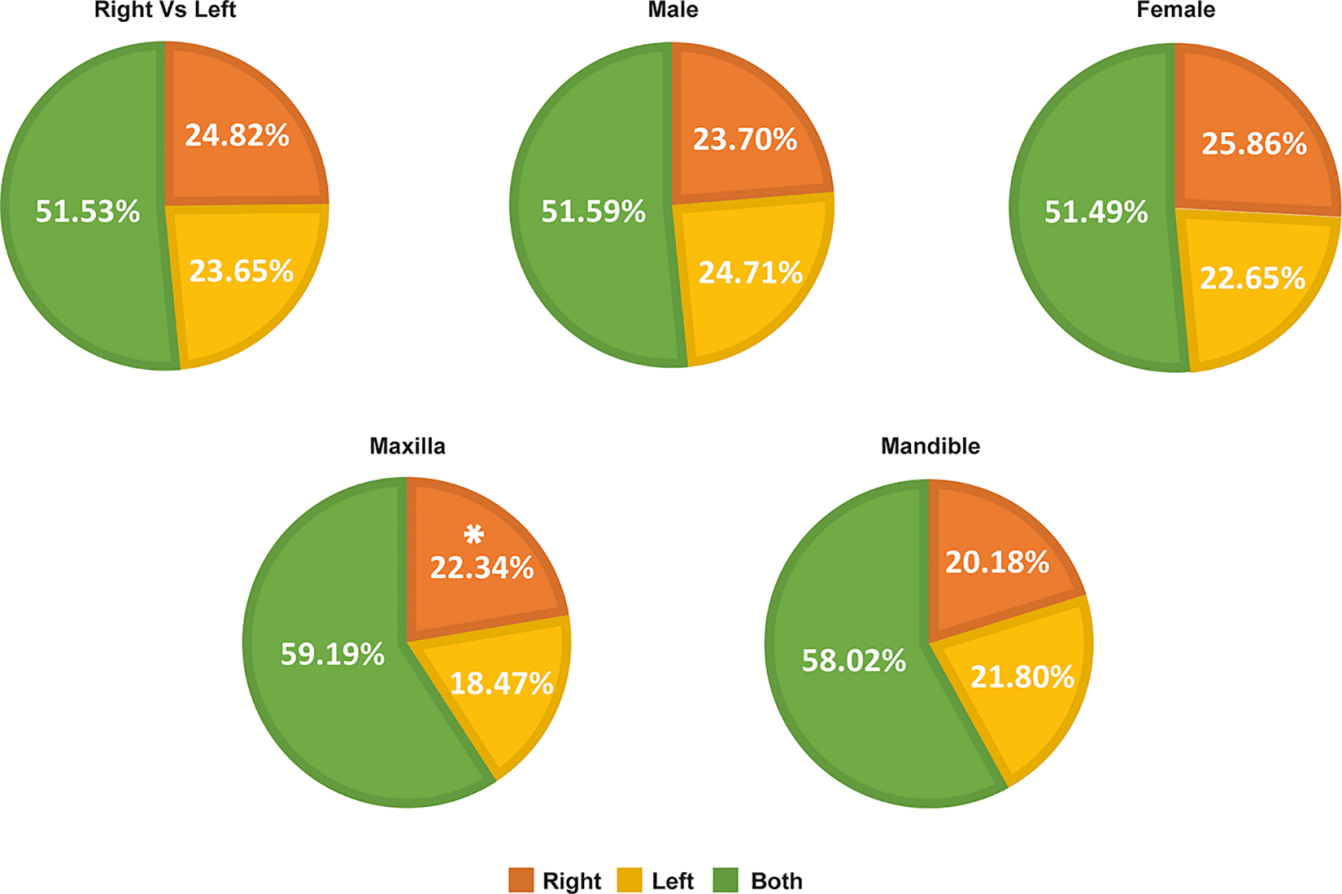
Distribution of agenesis according to side involvement.

Fig 2 represents the distribution of third molar agenesis according to side involvement. The difference between right and left side was not statistically significant. But in case of maxilla the frequency of third molar agenesis is higher in right side than left side (*p* value = .035).

### Other Dental Anomalies

Table 3 shows prevalence of other dental anomalies, which in total was 6.5%. Among them occurrence of hypodontia was 3.1%, hyperdontia was 0.7%, impacted canine was 1.6%, peg shaped lateral incisors were 0.1%, microdontia was 0.7% and dilacerations were 0.3%. Moreover, other dental anomalies were also compared with third molar presence group and third molar agenesis group. All the patients with agenesis were divided into 4 groups according to their third molar agenesis patterns: Group 1- Agenesis of 1 third molar (n = 685, Male-338 and Female- 347), Group 2- Agenesis of 2 third molars (n = 565, Male-277 and Female- 288), Group 3- Agenesis of 3 third molars (n = 174, Male-93 and Female- 81), Group 4- Agenesis of 4 third molars (n = 200, Male-111 and Female- 89) and Group 5- presence of third molars (n = 2604, Male-1351 and Female- 1253). Among the other dental anomalies microdontia was significantly higher in patients with agenesis (*p* = 0.015) and impacted canines were more commonly observed in patients without agenesis (*p* = 0.004). In addition, hypodontia was significantly higher in patients with agenesis of 3 third molars (p = 0.003).

**Table 3:** Distribution and comparison of the other dental anomalies in subjects with or without third molar agenesis.

	Third molar agenesis				Third molar agenesis Total	Third molar Presence Group	Total
	G1 (n = 685)	G2 (n = 565)	G3 (n = 174)	G4 (n = 200)	G1-4 (n = 1624)	G5 (n = 2604)	n = 4228
**Hypodontia**	20 (2.9)	19 (3.4)	12 (6.9)#	7 (3.5)	58 (3.6)	74 (2.8)	132 (3.1)
**Hyperdontia**	6 (0.9)	4 (0.7)	0 (0.0)	0 (0.0)	10 (0.6)	19 (0.7)	29 (0.7)
**Impacted Canine**	6 (0.9)	4 (0.7)	1 (0.6)	3 (1.5)	14 (0.9)	52 (2.0)*	66 (1.6)
**P. Lateral Incisor**	0 (0.0)	0 (0.0)	1 (0.6)	0 (0.0)	1 (0.1)	4 (0.2)	5 (0.1)
**Microdontia**	8 (1.2)	6 (1.1)	2 (1.1)	2 (1.0)	18 (1.1)*	12 (0.5)	30 (0.7)
**Dilaceration**	3 (0.4)	1 (0.2)	1 (0.6)	1 (0.5)	6 (0.4)	7 (0.3)	13 (0.3)
**Total anomalies**	43 (6.3)	34 (6.1)	17 (9.8)	13 (6.5)	107 (6.6)	168 (6.5)	275 (6.5)

P. Lateral Incisor- Peg Shaped Lateral Incisors

*Significant in Chi-square test and

# Significant in ANOVA.

## Discussion

**Fig 3:**
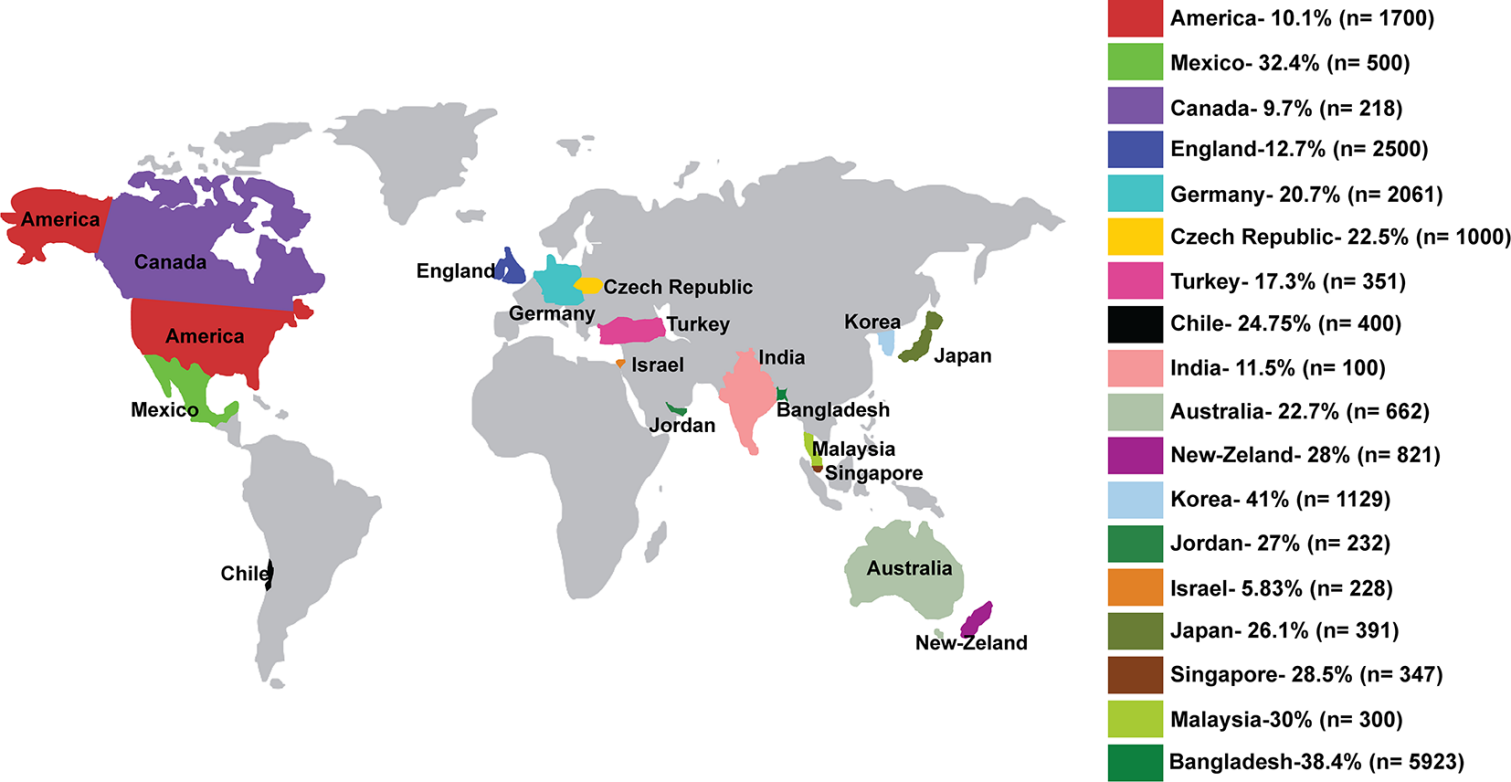
Global distribution of prevalence of third molar Agenesis.

In this study, our aim was to determine the prevalence of third molar agenesis from a large number (5923) of patients based on panoramic radiographs. According to Carter and Worthington [26], 2769 patients was the highest sample used so far. We investigated till date the largest sample size to determine prevalence of third molar agenesis in comparison with previous studies. Fig 3 shows global distribution of number of subjects and their prevalence of third molar agenesis.

In our study the minimum age was set at 10 years. Third molar crypt formation starts at 3 to 4 years of age. Calcification starts from 7 to 10 years and crown calcification completes at 12 to 16 years of age [27]. Racial variations, dietary habit, masticatory function and genetic inheritance can effect jaw size and facial growth, and this difference were easily noticeable among different studies of prevalence of third molar agenesis which were previously performed. In an animal study, Yamada and Kimmel [28] reported that diet and masticatory function had a direct relationship with craniofacial growth, specifically effecting the mandible, which could in turn affect the presence/agenesis of third molar. The prevalence of third molar agenesis in this study was 38.4% which is second highest in comparison with other populations. The lowest prevalence of third molar agenesis reported so far was 10.1% for African-Americans [29] and the highest prevalence was 41% for the Koreans [14]. Moreover, Kruger et al. [2] reported 28% for New-Zealanders, Malaysian Malays had 30% [11], and Indian Panjabi people had 11.5% [30] (Fig 3). It is interesting to note that the females presented a higher prevalence of third molar agenesis than males and these differences were statistically significant (p = .025). Our findings were in agreement with previous studies [2,14,31,32] but Alam et al. [11] reported that third molar agenesis was not influenced by sex. It can be explained by the sexual difference in craniofacial morphology. As the dimensions of dental arch of females are generally smaller than males. The maxillary and mandibular growth of females are slower after 12–13 years but the male growth continues until age of 16 years [33].

In this study the order of frequency for agenesis was 1 > 2 > 4 > 3. The order of frequency for the third molar agenesis in the present study differs from previous study by Celikoglu et al. [20], that reported an order of 1>2>3>4 in their findings. Sandhu and Kaur [30] and Jacob et al. [15] reported that third molar agenesis showed a greater predilection on maxilla than mandible which is in accordance with present study. In addition, present study didn’t find any significant predilection between right and left side. The side disparities in this study are in accordance with the results of Alam et al. [11]. Moreover, while checking for an association of side disparities with the sexual and intermaxillary differences, we found that third molar agenesis was more common on the right side of maxilla which was statistically significant. This unique finding has not been documented in the literature.

Another interesting feature of this study, overall prevalence of other dental anomalies was 6.5%, among those hypodontia was the highest (3.1%). Prevalence of hypodontia was significantly high in the patients with agenesis of 3 third molars (*p* = 0.003) and these findings are in agreement with Celikoglu et al. [34], who reported that hypodontia was frequently observed in patients with multiple third molars agenesis[35]. In addition, patients with agenesis group, microdontia was more frequent than the third molar presence group (*p* = 0.015). According to Garn and Lewis [36], patients with third molar agenesis had a general reduction in tooth size. Celikoglu et al. [34] also reported that patients with agenesis of 4 third molars had higher frequency of microdontia than patients without agenesis. Moreover, this study also found, impacted canine was more commonly observed in third molar presence group (*p* = 0.004) as compare to the third molar agenesis group.

Further efforts should be made to discern the etiology of third molar agenesis in order to refine our understanding of its variation among various populations. Genetic studies hold promise for deeper insight into this phenomenon. This study, will give the scientific community a more informed view of dental variations in humans.

## Conclusion

1. The present study reports 38.4% agenesis of third molar.
2. The frequency of third molar agenesis was found significantly greater in the females.
3. Third molar agenesis showed a greater predilection to maxilla in comparison to mandible.
4. These result revealed that among other dental anomalies, hypodontia had more prevalence.
5. While exploring other dental anomalies between third molar presence and agenesis group, microdontia in the agenesis group and impacted canine in the presence group were significantly more prevalent.
